# A Waning Superstition

**Published:** 1902-10-04

**Authors:** 


### A Waning Superstition.

Now that the small-pox epidemic by which London was threatened some months ago has practically passed away, interest tends to concentrate itself more especially upon the bill which will have to be paid for the extended hospital accommodation which has been forced upon the metropolis; and under these circumstances we may properly direct attention to the difficulties which are placed in the way of sanitary authorities in their efforts to supply isolation hospitals for small-pox patients, by the curious superstition as to the aërial convection of small-pox infection, which for 20 years or so has dominated every action of the Local Government Board in regard to this subject. So strongly does the Local Government Board hold to this theory that it requires that the site of a small-pox hospital must not have within a quarter of a mile of it either a hospital, workhouse, asylum, or any similar establishment, or a population of as many as 200 persons; nor must it have within half a mile of it a population of as many as 600 persons. Needless to say how enormously these requirements as to site add to the difficulties of providing hospitals for small-pox patients. In fact, many places find themselves in this absurd position: that while Parliament by its action in refusing to make vaccination effective has rendered the erection of hospitals necessary, the Local Government Board by its exactions as to site has rendered the erection of hospitals impossible.

Fortunately, the protests against this superstition are beginning to make themselves heard. We need not here enter upon any detailed criticism of the case of the Fulham Hospital, upon which the theory was built up; for that case never was a strong one, and in view of the general prevalence of the disease at the time, and especially of the number of persons who every day went in and out of the hospital gates, was quite beyond the range of proof. More to the point is it to bring forward cases in which, notwithstanding that hospitals have been established in contravention of the rules laid down by the Local Government Board, no harm has resulted. Dr. Scurfield, Medical Officer of Health for Sunderland, while lately criticising the official view in an address before the Society of Medical Officers of Health, brought forward the case of the small-pox hospital at the Sunderland Workhouse, a two-storied wooden structure in which, during the epidemic of 1883–4, upwards of 300 patients were treated. On the north of this building, at a distance of 30 feet, was the fever hospital, with usually some 20 to 30 inmates; farther north, at a distance of 144 feet, were the insane wards, with accommodation for 80. On the west, at a distance of 224 feet, was the school, containing 200 scholars. Nearly west, at a distance of 56 feet, was the lock hospital. On the north-west, at a distance of 136 feet, was the female side of the general hospital, with accommodation for 280. To the westward of the hospital were the buildings for the accommodation of the ordinary inmates, the portion of such buildings nearest to the small-pox hospital being at a distance of 150 feet. All the various sections of the workhouse mentioned were fully occupied while the small-pox hospital was in use, and the total population living on the place was about 900. The ambulance in bringing in the patients traversed a road between the house and the hospital; moreover, the wall round the wooden building, about 7 feet high, was at a distance varying from 10 to 22 feet from the building itself, and the children and adult inmates were able to come right up to the wall. Yet the medical officers of the workhouse were most emphatic in asserting that at no time during these two years did any spread of small-pox from the hospital to the neighbouring workhouse take place; and it is added that no attempt was made to protect the workhouse population by means of revaccination. During the last thirteen years nearly all the small-pox cases had, he said, been treated at the new General Hospital for Infectious Diseases, in a block surrounded by other buildings, and no spread of infection to the other pavilions had ever occurred. Dr. Reginald Green, Medical Officer for Gateshead, stated at the same meeting that he had been obliged to take small-pox cases into the General Hospital for Infectious Diseases, and had had as many as sixteen or seventeen cases in the wards usually occupied by typhoid-fever patients. But he had never had any spread to the rest of the hospital, nor had he any reason to think that the houses in the neighbourhood of the hospital had suffered from the circumstance. Among other instances which could be brought is the recent experience in London at the South Wharf Shelters, to which at least 8,000 cases have been conveyed on their way to the hospital ships. The shelters held 25 patients, and it is stated that on one occasion as many as 120 patients were detained there and on the steamboat when the traffic on the river was stopped by fog; yet there has been no spread of the disease in the neighbourhood of the shelters, their immediate vicinity indeed appearing to have been freer from small-pox than many other parts of the borough. The more we inquire into the management of small-pox hospitals the more convinced we are that when small-pox spreads in their neighbourhood this is due to personal rather than aërial conveyance of infection, and it is evident that the continued official maintenance of this old superstition as to aërial convection is a constant encouragement to laxity and to the diffusion of infection in other ways.

